# Changing trends in surgery for Graves’ disease: a cohort comparison of those having surgery intended to preserve thyroid function with those having ablative surgery

**DOI:** 10.1186/1916-0216-42-37

**Published:** 2013-05-29

**Authors:** Ahmed Al-Adhami, Ailsa C Snaith, Wendy L Craig, Zygmunt H Krukowski

**Affiliations:** 1 Department of General Surgery, Aberdeen Royal Infirmary, Aberdeen, UK; 2 Oxford Radcliffe Hospitals NHS Trust, Oxford, UK

**Keywords:** Graves’ disease, Hyperthyroidism, Thyroidectomy, Subtotal thyroidectomy, Total thyroidectomy

## Abstract

**Background:**

Surgery for Graves’ disease may be performed with the intent of preserving thyroid function (subtotal thyroidectomy) or ablating thyroid function (total thyroidectomy). This study examines the evolving practice in a specialist endocrine surgical unit.

**Method:**

Longitudinal cohort study of patients undergoing surgery for Graves’ disease between 1986 and 2008. Outcome measures were thyroid failure, recurrent toxicity, recurrent laryngeal nerve (RLN) palsy, early reoperation and hypocalcaemia. Time to thyroid failure was analysed by potential predictors.

**Results:**

Of 149 patients (129 female), 78 (52.3 percent) underwent subtotal thyroidectomy with the intention to preserve function (PF) and 71 (47.6 percent) total thyroidectomy with the intention to ablate thyroid function (AF). Mean duration of follow-up was 11.1 years; 14.8 years and 7.0 years respectively. Of 78 PF procedures: six (7.7 percent) patients suffered recurrent toxicity; 68 (87.2 percent) developed thyroid failure (four after treatment for recurrent toxicity); and eight (10 percent) remained euthyroid without replacement. Male gender and remnant gland weight were significant predictors of failure (P = 0.021 and 0.022 respectively). One patient developed permanent RLN palsy and one permanent hypocalcaemia. Of 71 AF procedures: one developed acute airway obstruction; one permanent RLN palsy; four permanent hypocalcaemia; and none developed recurrent toxicity. There were no deaths within a year of surgery. There was no statistically significant difference in complication rates.

**Conclusion:**

Most PF resections resulted in eventual thyroid failure. The shift to ablative surgery virtually eliminated the need for lifelong specialist follow-up, albeit with an insignificant rise in permanent hypocalcaemia.

## Background

Graves’ disease is the most common cause of hyperthyroidism affecting 0.5 percent of the population with a marked female preponderance [1]. There remains no consensus on the optimal management for patients with Graves’ disease. In contrast to practice in the United States, antithyroid medication rather than radio-active iodine is recommended as first line treatment in the UK and Europe [2]. Primary surgical management is uncommon and referral usually follows failure of medical therapy and radioiodine ablation or for specific indications including patient preference, pregnancy, and severe eye disease [2-4].

**Figure 1:**
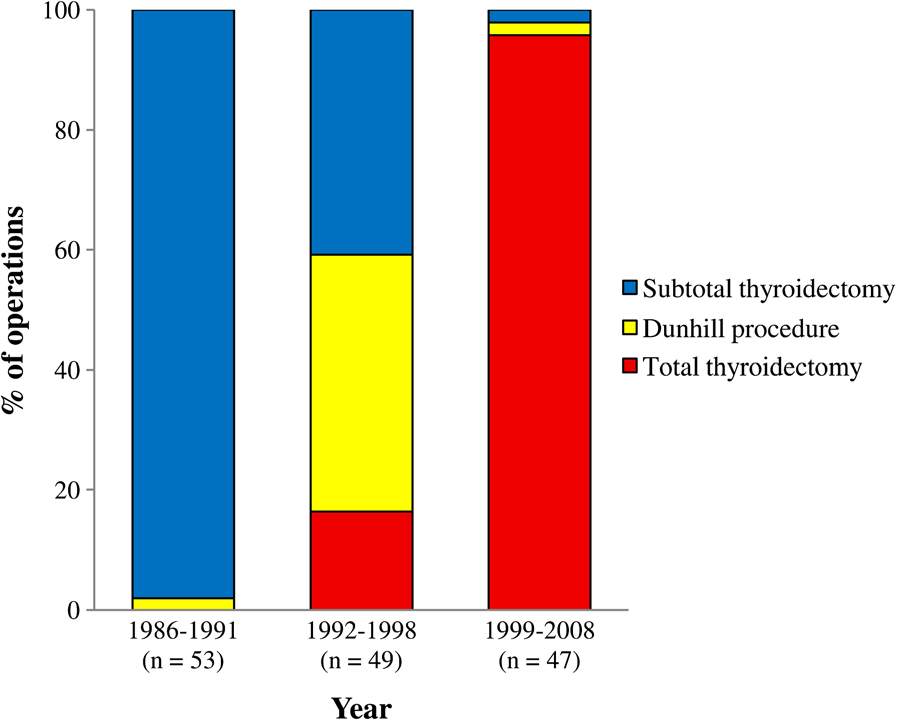
**Trends in surgery over time by type of thyroidectomy, 1986–2008.** Dunhill procedure (lobectomy and subtotal lobectomy), depending on remnant size, was used for both preservation and ablation of function.

Surgery is effective in the management of patients with Graves’ disease. It provides rapid control of hyperthyroidism and a high cure rate [3,5]. Two surgical options are available: subtotal thyroid resection aiming to preserve thyroid function and total thyroidectomy to ablate thyroid function eliminating the risk of recurrent toxicity and reoperation [3]. Before 1992, the policy in Aberdeen was to preserve thyroid function. Operations comprised subtotal thyroid resection preserving a thyroid remnant judged sufficient to permit recovery of normal thyroid function (bilateral subtotal lobectomy or lobectomy and contralateral subtotal lobectomy (Dunhill procedure)). After 1992, total thyroidectomy was progressively adopted becoming the operation of choice in 1999 (Figure 1).

Although total thyroidectomy has been associated with increased risk of complications [6], with increasing experience and better training, total thyroidectomy can be performed safely [7]. This study was designed to explore the impact of this change in policy on clinical outcome.

## Methods

This is a longitudinal cohort study of patients undergoing surgery for Graves’ disease in Aberdeen. Patients were under the care of two consultant surgeons with an interest in endocrine surgery. Data were collected *prospectively* on all patients between January 1986 and July 2008.

Surgical intervention was categorised according to the intention of the procedure whether to preserve (PF) or ablate thyroid function (AF). If the intent was to preserve function options included subtotal thyroidectomy or Dunhill Procedure. If ablation of function was the goal, total thyroidectomy or Dunhill procedure was used. Dunhill procedures were performed to preserve or ablate function depending on remnant size. On occasion a small remnant, insufficient to maintain euthyroidism, was preserved to protect the parathyroid glands. All patients were prepared for surgery using combinations of antithyroid drugs, beta-blockers and Lugol’s iodine.

Surgery was performed using the conventional technique [8]. The recurrent laryngeal nerve (RLN) was always identified and exposed. An attempt to identify and preserve all parathyroid glands was routine. The inferior thyroid artery was not routinely ligated to preserve parathyroid blood supply. If a gland was devascularised it was auto-transplanted in the sternomastoid muscle. During subtotal thyroidectomy, a large remnant (9–10 grams) was left if gland weight was judged to be more than 50 grams and a small remnant (2–4 grams) if less than 50 grams.

### Follow-up

All patients were reviewed at 1 month and then 6 monthly until euthyroid and normocalcaemic. Thereafter patients were registered on the Grampian Automated Thyroid Register (GAFUR) for automated biochemical and primary care review with recall to the endocrine service in the event of thyroid failure or relapse. Laryngoscopy was not routine until 2000 and only patients with voice change underwent laryngoscopy before this date.

### Outcomes

Outcome measures were thyroid failure, recurrent toxicity, RLN palsy, early reoperation and hypocalcaemia. Clinical thyroid failure was defined as clinical and biochemical evidence of hypothyroidism requiring thyroxine replacement. For patients undergoing surgery intended to preserve function, the time to thyroid failure was analysed by potential predictors. When ablative surgery was performed thyroxine replacement was commenced immediately. Recurrent toxicity was defined as clinical and biochemical evidence of thyrotoxicosis. Thyroid stimulating antibody (TSAb) measurements only became available later in the study.

### Statistical analysis

The Kaplan-Meier method was used to study the temporal relationship between surgery and thyroid failure and recurrent toxicity. Univariate analyses were performed using Fisher’s exact, Mann–Whitney *U* and log rank tests. A Cox regression analysis examined the potential association between baseline variables (including age, gender, resected and remnant weight) and subsequent thyroid failure. The likelihood ratios (LR) backward stepwise method was used with an entry criterion of P < 0.05 and a removal criterion of P > 0.10. P < 0.05 was considered statistically significant. Logistic regression was used to assess predictors of hypocalcaemia. Data were analysed using SPSS for Windows version 16.0 (SPSS, Chicago, Illinois, USA).

## Results

### Demographics and baseline characteristics

Between 1986 and 2008, there was a gradual reduction in the number of operations performed and a total of 149 patients underwent surgery for Graves’ disease (Figure 1). Patients comprised 129 females (86.5 percent) and 20 males (13.4 percent) with a median age of 30 years (Interquartile range (IQR) 20–34, range 12–65 years) (Table 1). Of 149 patients, 10 (6.7 percent) were referred for surgery without a previous course of antithyroid therapy - patient preference (50 percent) was the commonest indication for this approach. One hundred and thirty-nine patients (93.3 percent) were referred after a trial of one or more courses of antithyroid drugs. In these patients relapse (46.0 percent), inadequate control of thyrotoxicosis (38.1 percent) and patient preference (10.8 percent) were the commonest indications for surgical referral.

**Table 1:** Baseline characteristics

**Characteristics**		**Intention to preserve function (n = 78)**	**Intention to ablate function (n = 71)**
**Age, median (range), years**		27 (17–54)	32 (12–65)
**Female**		66 (84.6)	63 (88.7)
Indication(s) for surgery*	Relapse	32 (41.0)	32 (45.1)
	Inadequate control of thyrotoxicosis	18 (23.1)	35 (49.3)
	Patient preference	8 (10.3)	19 (26.8)
	Poor compliance with ATD	15 (19.2)	7 (9.9)
	Large goitre	7 (9.0)	5 (7.0)
	Severe thyroid eye disease	1 (1.3)	3 (4.2)
	Other**	5 (6.4)	12 (16.9)
**Operation**	Subtotal thyroidectomy or Dunhill procedure	78 (100.0)	18 (25.4)
	Total thyroidectomy	NA	53 (74.6)
**Gland weight**	Total gland weight, mean (IQR), grams	62 (46–78)	50 (37–64)
	Remnant gland weight, mean (IQR), grams	7 (5–8 )	1 (0–1)
	Resected gland weight, mean (IQR), grams	55 (42–70)	50 (36–64)
	Proportion of gland resected (%)	90 (88–92)	100 (98–100)
**Follow-up, mean (IQR), years**		14.8 (12.1–19.4)	7.0 (3.2–10.8)

Values in parentheses are percentages unless otherwise indicated. *NA* not applicable. *ATD* antithyroid drugs. *IQR* Interquartile range. *Some patients fulfilled more than one indication for surgery. ** Pregnancy, adverse drug reactions and failure of radioiodine.

Of 149 patients, 73 (49.0 percent) underwent a subtotal thyroidectomy, 23 (15.4 percent) a Dunhill procedure, and 53 (35.6 percent) a total thyroidectomy (Table 1). In 78 patients (52.3 percent) the intent was to preserve normal function (PF group). In the remaining 71 patients (47.7 percent) the intent was to ablate function with immediate thyroxine replacement (AF group). Mean duration of follow-up was 11.7 years (IQR 4.7 to 16.9 years, range 0.1 to 22.5 years). There was no difference in gender distribution between the two groups (Table 1). The estimated total gland weight ranged from 10 to 240 g (median 57 g, IQR 41–74 g) (Table 1). Histologically, all 149 specimens showed evidence of Graves’ disease and one (0.7 percent) specimen an incidental papillary carcinoma greater than 1 cm.

### Time to failure with PF surgery

**Figure 2:**
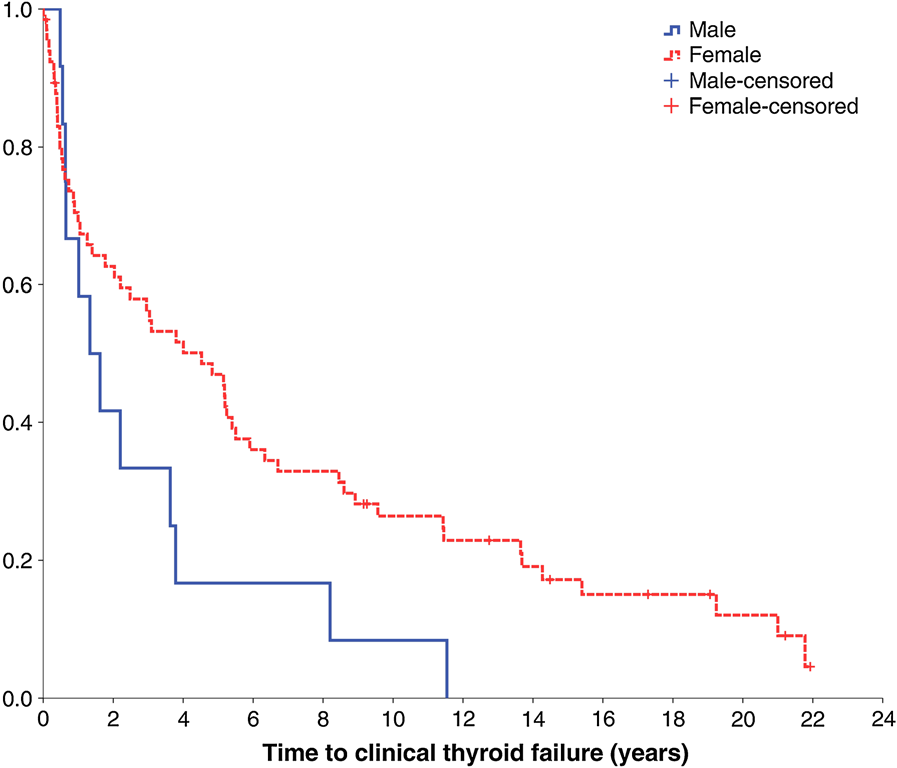
Time to thyroid failure after PF surgery by sex.

Of the 78 patients undergoing PF surgery, 66 (84.6 percent) were female and 12 (15.4 percent) were male. Sixty-eight (87.2 percent) were subsequently commenced on thyroxine replacement (Figure 2 Additional file 1: Table S1); four (5.1 percent) of them after radioiodine treatment for recurrent toxicity.

On Cox regression analysis, gender (P = 0.021) and remnant weight (P = 0.022) were independent predictors of thyroid failure with hazard ratios of 2.197 and 0.713 respectively. Males were associated with a significantly higher rate of failure over time (Figure 2 Additional file 1: Table S1). Age at operation (P = 0.593), duration of diagnosis prior to surgery (P = 0.668), resected weight (P = 0.298) and percentage of gland resected (P = 0.267) did not significantly predict thyroid failure.

At five years, a higher rate of thyroid failure was observed in patients under 30 years; however, at subsequent follow-up beyond five years, no difference was observed (*X*^2^ = 0.03, P = 0.874; log rank test). When compared with glands weighing more than 50 grams, patients with glands of 50 grams or less were more likely to develop thyroid failure earlier over time (*X*^2^ = 6.76, P = 0.009; log rank test).

Resected specimens were routinely examined for evidence of lymphocytic infiltration. Lymphocytic infiltration was ‘absent’ or not noted in 46 (59.0 percent) specimens, ‘minimal’ in 21 (26.9 percent) specimens, and ‘marked’ in 11 (14.1 percent) specimens. Although not statistically significance (*X*^2^ = 3.57, P = 0.059; log rank test), a higher rate of early failure was observed in patients with ‘marked’ lymphocytic infiltration.

### Recurrent toxicity

Of the 78 PF operations, six (7.7 percent), all female, developed recurrent toxicity; 3 at one year, 5 at five years and 6 at 11 years. Recurrence was treated with radioactive iodine and antithyroid drugs. Although not statistically significant, recurrent toxicity was commoner among patients over 30 years (3 of 20 versus 3 of 58; *X*^2^ = 1.86, P = 0.173), total gland weights over 50 grams (5 of 55 versus 1 of 23; *X*^2^ = 0.58, P = 0.448), and gland resections of 90 percent or less (4 of 40 versus 2 of 38; *X*^2^ = 0.50, P = 0.479). Recurrence was not encountered in patients undergoing thyroid ablation.

### Morbidity

There were no deaths within a year of surgery. One patient (0.7 percent) developed acute airway obstruction requiring urgent evacuation of haematoma (Table 2). Seven (4.7 percent) patients had recurrent laryngeal nerve (RLN) palsy confirmed by laryngoscopy; two (1.3 percent) of them permanent as a result of intraoperative injury or traction. None developed bilateral palsy. The most common early complication was transient hypocalcaemia (serum calcium less than 2.2 mmol/l) occurring in 87 (58.4 percent) patients; highest after total thyroidectomy (84.9 percent) followed by the Dunhill procedure (65.2 percent) and subtotal thyroidectomy (37.0 percent). Hypocalcaemia was permanent in 5 (3.4 percent) patients; highest after total thyroidectomy (7.5 percent) and lowest after Dunhill procedure (0.0 percent). Crude analyses revealed an increased risk of transient hypocalcaemia with total thyroidectomy (Table 3). On logistic regression analysis, the type of thyroidectomy constituted an independent risk factor for transient but not permanent hypocalcaemia. Age, gender and number of parathyroid glands seen, preserved or reimplanted did not affect the onset of transient or permanent hypocalcaemia.

**Table 2:** Postoperative complications and hospital stay

	**Intention to preserve (n = 78)**	**Intention to ablate (n = 71)**	
	**No. (%)**	**No. (%)**	P value*
Acute airway obstruction**	0 (0.0)	1 (1.4)	0.477
**Subcutaneous haematoma**	1 (1.3)	1 (1.4)	1.000
**Transient RLN palsy†**	1 (1.3)	4 (5.6)	0.192
**Permanent RLN palsy†**	1 (1.3)	1 (1.9)	1.000
**Permanent hypocalcaemia††**	1 (1.3)	4 (5.6)	0.192
**Wound infection**	4 (5.1)	2 (2.8)	0.683
**Wound seroma**	0 (0.0)	1 (1.4)	0.477
**Total hospital stay, mean (SD) (median), days**	3.30 (1.43) (3.0)	2.18 (1.11) (2.0)	0.003‡
**Recovery time, mean (SD) (median), days**	2.83 (1.44) (2.0)	1.87 (1.43) (1.0)	0.005‡

Values in parentheses are percentages unless specified. *RLN*, recurrent laryngeal nerve.

* Fisher’s exact test unless specified.

** Airway obstruction due to deep tension haematoma.

† Laryngoscopy was not routine until 2000 and only patients with voice change underwent laryngoscopy before this date.

†† Relative risk (95% CI) comparing intention to ablate with intention to preserve = 4.39 (0.50–38.9).

‡Mann–Whitney *U* test.

**Table 3:** Rates of postoperative hypocalcaemia

	**No./total (%)**	Relative risk (95% CI)^*^
**Hypocalcaemia, transient or permanent**		
Subtotal thyroidectomy or Dunhill procedure	43/96 (44.8)	1 [Reference]
Total thyroidectomy	49/53 (92.5)	2.06 (1.63–2.61)
**Transient hypocalcaemia**		
Subtotal thyroidectomy or Dunhill procedure	42/73 (43.8)	1 [Reference]
Total thyroidectomy	45/53 (84.9)	1.94 (1.51–2.50)
**Permanent hypocalcaemia**		
Subtotal thyroidectomy or Dunhill procedure	1/96 (1.0)	1 [Reference]
Total thyroidectomy	4/53 (7.5)	7.26 (0.83–63.17)

Values in parentheses are percentages.

^*^ Relative risks compare total thyroidectomy with subtotal thyroid resection (subtotal thyroidectomy or Dunhill procedure).

Table 2 stratifies complications by operative intent. Total hospital stay and recovery time (time from operation to discharge) were significantly shorter for patients undergoing ablative surgery (P = 0.003 and 0.005 respectively; Mann–Whitney *U* test) (Table 2). The difference in length of hospital stay is likely to reflect a progressive change in NHS policy towards early discharge.

## Discussion

The management of Graves’ disease aims to control hyperthyroidism and restore euthyroidism. With the advent of radioiodine ablation, a gradual reduction in the rate of surgery was observed. In 1972, Michie et al. [9] reported an annual rate of 56 operations for thyrotoxoicosis in Aberdeen. By 1987, Cusick et al. [4] reported 16 operations per annum and in 1999 this declined to 8 operations per annum [10]. This trend continues despite an apparently stable incidence of disease.

The commonest indications for surgery were inadequate control or repeated relapse. The aim of surgery in Graves’ disease is to provide a rapid and permanent means of controlling hyperthyroidsim. Subtotal thyroidectomy was the standard treatment for Graves’ disease for most of the twentieth century. This approach aims to restore function and reduce complications by preserving a remnant of thyroid tissue [6,11]. Palit et al. [5] reported that subtotal thyroidectomy achieved euthyroidism in 60 percent of patients and supported its use in milder forms of hyperthyroidism.

Before 1992, the surgical policy in Aberdeen was to perform subtotal resections with a view to preserving thyroid function. Such policy was based on previous experience suggesting higher rates of thyroid failure with smaller remnants [9]. Subsequent evidence [4] revealed that recurrence was greatest in small glands. After 1992, smaller remnants of 2–4 grams were preserved in small glands to avoid recurrent toxicity albeit at the cost of a higher rate of thyroid failure; and larger remnants of 9–10 grams in large glands to avoid thyroid failure. As practice evolved, and considering published evidence, the policy moved towards routine total thyroidectomy (Figure 1).

There are only two prospective randomised studies [6,12] comparing total and subtotal thyroid resection and neither followed consolidated standards of reporting trials (CONSORT). Both studies focused primarily on the impact of surgery on thyroid eye disease and concluded subtotal resection was the optimum procedure. Although early complication rates were provided, maximum follow-up times were under five years. The incidence of thyroid failure following subtotal resection was not investigated or relevant because small remnants were preserved with the intent of ablating function. The present study represents the first in-depth prospective analysis of time to thyroid failure following subtotal resection with the intent to preserve function. This long-term study demonstrates that thyroid failure can occur at 22 years and recurrent toxicity beyond 10 years.

A myriad of factors including remnant weight, degree of lymphocytic infiltration, and thyroid stimulating hormone receptor, thyroid peroxidase or antithyroglobulin antibodies for their value in predicting function after subtotal thyroidectomy [2,4,9,11,13,14]. Most studies concluded that remnant gland weight is the most consistent risk factor. This study investigated the incidence of thyroid failure over time in patients undergoing surgery intended to preserve thyroid function (mainly subtotal thyroidectomy). The prevalence of thyroid failure in this cohort exceeds other prevalence estimates although this may be related to the length of follow-up [5,11,14]. Female patients and those with higher remnant weights demonstrated longer periods of euthyroidism but the majority developed clinical thyroid failure within the first decade. Our finding that gender and the amount of residual tissue influence the risk of failure supports previously published data [4,5,11]. However in contrast to previous studies [4,11], the relationship between age and thyroid failure was not statistically significant.

Recurrent toxicity is a more significant complication than thyroid failure which can almost be considered the end point in most patients. Although early complication rates after operations to preserve function are minimal, there is a risk of recurrence. Reoperations are technically more difficult and carry higher risks of morbidity as a result [15]. This study had a recurrence rate of 7.7 percent with the majority in the five years following surgery. PF operations increase the risk of undetected hypo or hyperthyroidism and reoperation requiring lifelong follow-up [14].

After the introduction of total thyroidectomy, the risk of recurrent toxicity was eliminated sparing complications of reoperation and radioiodine ablation [1,15]. Total thyroidectomy coupled with immediate thyroxine replacement achieves a rapid and permanent return of euthyroidism. A recent study from the United States [16] showed that total thyroidectomy is more cost-effective than radioiodine or lifelong antithyroid medication in patients failing to achieve euthyroidism after 18 months of medical treatment by avoiding the need for lifelong surveillance.

Hypocalcaemia and recurrent laryngeal nerve (RLN) palsy are well recognised complications of thyroid and parathyroid surgery. Previous studies have presented comparable complication rates between subtotal thyroidectomy and total thyroidectomy in Graves’ disease [2]. In a meta-analysis of 35 clinical studies between 1965 and 1998, Palit et al. [5] reported no significant difference in the rate of either permanent recurrent laryngeal nerve palsy (0.7 percent of subtotal thyroidectomy and 0.9 percent of total thyroidectomy) or permanent hypocalcaemia (1.0 and 0.9 percent respectively) between subtotal and total thyroidectomy.

Ablative surgery exposed patients to a significantly higher rate of transient, but not permanent, complications. In an analysis of 5846 patients undergoing bilateral thyroid surgery, Thomusch et al. [17] identified Graves’ disease, total thyroidectomy, bilateral central ligation of the inferior thyroid artery, and identification and preservation of no or only a single parathyroid gland as independent risk factors for permanent hypocalcaemia. Our study concluded an overall incidence of 58.4 percent for transient hypocalcaemia increasing significantly with more extensive resections. The overall occurrence of permanent hypocalcaemia was 3.4 percent; this was more frequently observed with total thyroidectomy but statistically insignificant. However, given the small sample size in this study, the possibility of a type II error cannot be excluded.

RLN palsy, mainly transient, is the second most common complication following thyroid surgery. The incidence varies depending on the underlying disease, the extent of resection, whether first-time or reoperation, and the surgical experience and technique [18,19]. Routine RLN identification was performed in all patients. The third national audit of the British Association of Endocrine and Thyroid Surgeons (BAETS) documented an overall RLN palsy rate of 2.5 percent for first-time operations [20]. Studies in Graves’ disease however, have reported rates of permanent palsy ranging from 0 percent to as high as 4.5 percent [4,21]. In this study, permanent RLN palsy occurred in 2 (1.3 percent) patients with no significant difference between groups. Laryngoscopy was not routine prior to 2000 and only undertaken in the presence of voice change; it is therefore possible that a permanent palsy could have been overlooked.

It is known that resected specimens from patients with Graves’ disease exhibit an increased incidence of incidental carcinomas [22]. When collating many studies, Palit and colleagues [5] reported a rate of 4.5 percent. However, despite a worldwide increase in thyroid cancer and regional trends of advanced disease [23], the incidence of carcinoma greater than 1cm, in this cohort was low (0.7 percent) in spite of careful review by a dedicated pathologist in a multidisciplinary setting. Microcancers were not recorded in the prospective database.

The strengths of this study include its prospective design and long duration of follow-up. All patients were under the care of only two consultant surgeons reducing perioperative variability. The limitations of this study include a relatively small sample size with the possibility of a Type II error and its nonrandomised nature with the likelihood of selection bias. However, since the study monitors the effect of a known change in surgical practice on all patients, the degree of selection bias is minimal.

The perceived advantages of subtotal thyroidectomy were reduced complication rate and possibility of maintaining euthyroidism. However this study concurs with the growing evidence that complications of total thyroidectomy are no different and subsequent management is greatly simplified. With prolonged follow-up it is clear that thyroid failure is likely in the majority of patients treated surgically and the primary goal of treatment should change from avoidance of hypothyroidism to prevention of recurrence.

## Conclusions

Results support previous studies demonstrating low complication rates with thyroid surgery. The majority undergoing PF resections eventually developed thyroid failure. AF resections eliminate the need for lifelong specialist follow-up by abolishing the risk of recurrent toxicity and undetected hypothyroidism, albeit with an insignificant rise in permanent hypocalcaemia.

## Abbreviations

PF: Surgery intended to preserve thyroid function; AF: Surgery intended to ablate thyroid function; LR: Likelihood ratio; ATD: Antithyroid drugs; IQR: Interquartile range; RLN: Recurrent laryngeal nerve; CI: Confidence interval.

## Competing interests

The authors declare that they have no competing interests.

## Authors’ contributions

AA contributed to study design, data collection, analysis and manuscript drafting. ACS contributed to data collection and study design. WLC contributed to data analysis and manuscript drafting. ZHK conceived the study and contributed to study design, data analysis and manuscript drafting. All authors read and approved the final manuscript.

## Authors’ information

AA and ACS are junior surgical trainees. WLC is a senior surgical trainee with specialist interest in endocrine surgery. ZHK is the professor of clinical surgery and a consultant surgeon with specialist interest in endocrine surgery.

## Supplementary Material

Additional file 1: Table S1 Number at risk table. Click here for file
