# Supplementary material for: Changing trends in surgery for Graves’ disease: a cohort comparison of those having surgery intended to preserve thyroid function with those having ablative surgery
Source: J Otolaryngol Head Neck Surg. 2013 May 29;42(1):37. doi: 10.1186/1916-0216-42-37 (PMC3681644; doi:10.1186/1916-0216-42-37)
Supplement: Additional file 1: Table S1 — Number at risk table. [file 1916-0216-42-37-S1.doc]

Supplement to Figure 2 – Number at risk table

|  |  | **0** | **2** | **4** | **6** | **8** | **10** | **12** | **14** | **16** | **18** | **20** | **22** |
| --- | --- | --- | --- | --- | --- | --- | --- | --- | --- | --- | --- | --- | --- |
| **Number at risk** | **Male** | 12 | 5 | 2 | 2 | 2 | 1 | 0 | 0 | 0 | 0 | 0 | 0 |
|  | **Female** | 66 | 40 | 33 | 23 | 21 | 15 | 13 | 10 | 7 | 6 | 4 | 0 |
